# Relationship of Circulating Fetuin-A Levels with Body Size and Metabolic Phenotypes

**DOI:** 10.1155/2018/7918714

**Published:** 2018-12-24

**Authors:** Hye Soo Chung, Hyun Jung Lee, Soon Young Hwang, Ju-Hee Choi, Hye Jin Yoo, Ji A. Seo, Sin Gon Kim, Nan Hee Kim, Dong Seop Choi, Sei Hyun Baik, Kyung Mook Choi

**Affiliations:** ^1^Division of Endocrinology and Metabolism, Department of Internal Medicine, Kangnam Sacred Heart Hospital, Hallym University College of Medicine, Seoul, Republic of Korea; ^2^Division of Endocrinology and Metabolism, Departments of Internal Medicine, Korea University, Seoul, Republic of Korea; ^3^Department of Biostatistics, College of Medicine, Korea University, Seoul, Republic of Korea

## Abstract

**Background:**

Previous studies have suggested the existence of distinct body size subgroups according to metabolic health referred to as metabolically healthy obesity (MHO) and metabolically abnormal but normal weight (MANW) patients. Although nonalcoholic fatty liver disease (NAFLD) is strongly associated with obesity and metabolic syndrome, the relationship between these phenotypes and fetuin-A, a representative hepatokine, has not been explored.

**Methods:**

We examined the association between circulating fetuin-A levels, metabolic health phenotypes, cardiometabolic risk parameters, and subclinical atherosclerosis in 290 subjects who were randomly selected from an ongoing cohort study.

**Results:**

Fetuin-A concentrations were significantly associated with detrimental anthropometric and laboratory measurements, including increased waist circumference, blood pressure, alanine aminotransferase, fasting plasma glucose, and triglyceride levels. Furthermore, fetuin-A levels were significantly increased in the metabolically abnormal (MA) group compared to the metabolically healthy (MH) group in subjects without obesity (717.1 [632.1, 769.7] vs. 599.5 [502.0, 709.3], *P* = 0.001) and subjects with obesity (704.1 [595.5-880.9] vs. 612.2 [547.9-802.1], *P* = 0.016). In addition, brachial-ankle pulse wave velocity (baPWV), which reflects arterial stiffness, was higher in MA individuals compared to MH individuals. Multiple logistic regression analysis revealed that both individuals without obesity (*P* for trend = 0.017) and with obesity (*P* for trend = 0.028) in the higher tertiles of fetuin-A had an increased risk of MA than those in the lowest tertile.

**Conclusions:**

This study demonstrates that fetuin-A levels are significantly associated with metabolic health phenotypes, such as MHO and MANW, in Korean adults.

## 1. Introduction

Emerging evidence suggests the existence of a subset of individuals with obesity in the absence of the metabolic and cardiovascular sequelae of obesity known as “metabolically healthy obesity” (MHO) [1, 2]. On the contrary, some individuals have an increased burden of these risks despite having normal weight, which is termed “metabolically abnormal but normal weight” (MANW) [3]. We reported that elderly MANW subjects had increased all-cause mortality compared to those with the MHO phenotype during 10 years of follow-up in the South-West Seoul (SWS) study [4]. A comprehensive understanding of the pathophysiological mechanisms of MHO and MANW may provide insight about individualized interventions and may reduce health care costs for patients with obesity and/or metabolic disorders.

Prior studies reported that various underlying mechanisms such as insulin resistance, inflammation, hormonal changes, and physical activity can determine whether a person with obesity is metabolically healthy or not [5, 6]. We previously found an association between low muscle mass and metabolic health phenotypes, including MHO and MANW, in the Korean Sarcopenic Obesity Study (KSOS) [7]. Nonalcoholic fatty liver disease (NAFLD), which is a hepatic manifestation of metabolic syndrome, is closely associated with obesity [8]. Stefan et al. found that ectopic fat in the liver might be a more important determinant than visceral fat for a beneficial phenotype in obesity [9]. In our previous study, only decreased levels of liver enzymes were independently associated with the MHO phenotype, even though both liver enzymes and vitamin D levels are related to insulin resistance and metabolic syndrome [10]. Messier et al. also reported that lower levels of liver enzymes could be involved in the protective profile of MHO individuals [11]. Heianza et al. showed that almost half of MHO subjects had ultrasonographic fatty liver in a Japanese population [12]. They found that patients in the MHO group with fatty liver had an elevated risk of diabetes (odds ratio (OR) = 4.09 (95% CI = 2.20-7.60)), whereas the risk of diabetes in the MHO group without fatty liver was not significantly increased (OR = 1.01 (95% CI = 0.35-2.88)) [12]. These results suggest that ectopic liver fat and hepatic function may have a critical role in metabolic health.

Recent research has demonstrated that a group of predominantly liver-derived proteins called hepatokines influence glucose and lipid metabolism in a similar way as adipokines and myokines [13]. Fetuin-A is a representative hepatokine that induces insulin resistance in rodents [14] and is positively associated with insulin resistance and hepatic steatosis in humans [15, 16]. Ix et al. reported that fetuin-A levels are associated with incident diabetes independent of other confounding factors, including insulin resistance, during a 6-year follow-up [17]. Although fetuin-A has been suggested to be an important link between NAFLD and metabolic disorders, to the best of our knowledge no previous studies have explored the relationship between fetuin-A and metabolic health phenotypes.

In the present study, we examined the relationship of fetuin-A in MHO and MANW individuals along with cardiometabolic risk factors and subclinical atherosclerosis in Korean men and women without a history of cardiovascular disease (CVD).

## 2. Material and Methods

### 2.1. Study Design and Participants

This ongoing longitudinal study was designed to examine the prevalence of metabolic health phenotype and its impact on health outcomes in Korean adults [18, 19]. Participants were recruited based on predefined inclusion and exclusion criteria from individuals who were self-referred for a routine health check-up at the Health Promotion Center of Korea University Guro Hospital between April 2012 and July 2014, and a follow-up survey has been in progress since 2015. Participants were apparently healthy Korean men and women between 40 and 80 years old, residing in Seoul, South Korea. Subjects were excluded from this study if they met any of the following criteria: history of CVD (myocardial infarction, unstable angina, stroke, or cardiovascular revascularization); stage 2 hypertension (resting blood pressure, ≥160/100 mmHg); malignancy; a positive test for hepatitis B surface antigen or hepatitis C antibody; use of herbal medication or medications that might affect inflammatory status, including steroid and nonsteroidal anti-inflammatory within 6 months; or severe renal or hepatic disease. Medical histories and lifestyle information including smoking and alcohol consumption were collected for all subjects by personal interview using a detailed questionnaire. Alcohol consumption was categorized into three groups: “never,” “current,” and “ex-drinker.” An ex-drinker was defined as someone who used to drink but had not had any alcohol at that time. In terms of smoking, individuals were also classified as “never,” “current,” or “ex-smokers.” Among 1012 participants, 290 subjects were randomly selected, and circulating fetuin-A concentrations were measured for this study. All participants provided written informed consent, and the Korea University Institutional Review Board approved the study protocol in accordance with the Declaration of Helsinki of the World Medical Association.

### 2.2. Anthropometric and Laboratory Measurements

Body mass index (BMI) was calculated as weight/height^2^ (kg/m^2^), and waist circumference was measured at the midpoint between the lower border of the rib cage and the iliac crest. All blood samples were obtained in the morning after a 12-hour overnight fast and were immediately stored at −80°C for subsequent assays. Total cholesterol, high-density lipoprotein (HDL) cholesterol, low-density lipoprotein (LDL) cholesterol, triglycerides, and aspartate aminotransferase (AST)/alanine aminotransferase (ALT) were measured using an automatic biochemical analyzer (TBA-2000FR; Toshiba Medical Systems, Japan). The glucose oxidase method was used to measure fasting plasma glucose (FPG) levels, and a latex-enhanced turbidimetric immunoassay (HiSens hsCRP LTIA; HBI, Anyang, Korea) was used to measure high-sensitive C-reactive protein (hsCRP). Kidney function was determined from the estimated glomerular filtration rate (eGFR) calculated with the Chronic Kidney Disease Epidemiology Collaboration (CKD-EPI) formula [20]. Serum fetuin-A levels were assayed using a commercially available ELISA (R&D systems, Minneapolis, USA), and the intra- and interassay CVs were 3.9–4.9% and 7.4–8.4%, respectively.

### 2.3. Definitions of Metabolic Health Phenotypes

Obesity was defined according to the criteria recommended by the Korean Society for the Study of Obesity, which defines “normal” as a BMI ≥18.5 and <25.0 kg/m^2^ and “obesity” as a BMI ≥25.0 kg/m^2^ [8]. Metabolic syndrome (MetS) was defined according to the criteria established by the National Cholesterol Education Program Adult Treatment Panel III using the adjusted waist circumference for Asians [21, 22]. By combining the BMI and MetS groups, all study subjects were classified into four groups: (i) normal weight without MetS (metabolically healthy normal weight; MHNW), (ii) normal weight with MetS (metabolically abnormal but normal weight; MANW), (iii) obesity without MetS (metabolically healthy obesity; MHO), and (iv) obesity with MetS (metabolically abnormal obesity; MAO).

### 2.4. Measurement of Carotid Intima-Media Thickness (CIMT) and Brachial-Ankle Pulse Wave Velocity (baPWV)

The intima-media thickness (IMT) of the common carotid artery was determined using a high-resolution B-mode ultrasonography (EnVisor; Philips Healthcare, Andover, MA, USA) with a 5 to 12 MHz transducer. Measurements of CIMT were made using measurement software (Intimascope; Media Cross Co., Tokyo, Japan) at three levels of the far wall, which is 1~3 cm proximal to the carotid bifurcation. The mean CIMT was the average value of 99 computer-based points in the region, and the maximal CIMT was defined as the IMT value at the maximal point in the region. The intraobserver variability coefficient of CIMT was 0.93. After a subject rested in the supine position for 5 min, baPWV was measured using a BP-203RPE II volume-plethysmographic apparatus (Colin, Komaki, Japan). Brachial and ankle blood pressure on the left and right sides and baPWV values were calculated as the mean of the left and right baPWV values. Methodological details, including validity and reproducibility, have been described in previous studies [23].

### 2.5. Statistical Analyses

Data are expressed as means ± standard deviations, medians and interquartile ranges (25%-75%), or number (percentages). Differences between two groups were identified using Student's *t*-test or the Mann-Whitney *U*-test, and the *χ* ^2^-test was used to assess differences in the distribution of categorical variables. The partial Spearman's correlation coefficient adjusted for sex and age was used to evaluate the correlations of serum fetuin-A levels with metabolic risk factors. Multiple logistic regression analysis was performed to identify whether tertiles of circulating fetuin-A levels could influence the risk of metabolic syndrome even after adjusting for other confounding factors in groups with and without obesity, respectively. Each variable was examined to ensure a normal distribution. A *P* value < 0.05 was considered statistically significant in all analyses. All statistical results were based on two-sided tests. Data were analyzed using SAS for Windows (version 9.20, SAS Institute Inc., Cary, NC, USA).

## 3. Results

### 3.1. Characteristics of the Study Subjects

**Figure 1:**
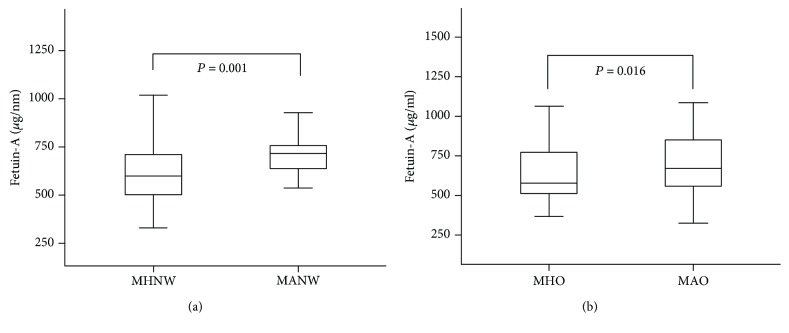
Difference in circulating fetuin-A concentrations in subjects without obesity (a) and with obesity, (b) based on their metabolic health phenotype. MHNW: metabolically healthy normal weight; MANW: metabolically abnormal but normal weight; MHO: metabolically healthy obesity; MAO: metabolically abnormal obesity.

Table 1 shows the characteristics of study participants based on obesity and metabolic health phenotype. As expected, in both subjects with and without obesity, most of the metabolic syndrome components were significantly different between the metabolically healthy (MH) group and the metabolically abnormal (MA) group. Furthermore, ALT levels in all study participants and AST levels in the obesity group showed significant differences according to metabolic health phenotype. In particular, fetuin-A concentrations were increased in the MA group compared to the MH group in subjects without obesity (717.1 [632.1, 769.7] vs. 599.5 [502.0, 709.3], *P* = 0.001) and with obesity (704.1 [595.5-880.9] vs. 612.2 [547.9-802.1], *P* = 0.016) (Figure 1). Interestingly, MA individuals had higher baPWV levels than MH individuals in both nonobesity and obesity, although CIMT levels were not different.

### 3.2. Correlation Analysis between Fetuin-A Concentrations and Various Cardiometabolic Risk Parameters

In Table 2, Spearman correlation analysis demonstrates the relationship between circulating fetuin-A levels and cardiometabolic risk variables. In all study participants, fetuin-A levels were positively associated with BMI, waist circumference, blood pressure, ALT, FPG, and triglyceride levels and were negatively associated with total bilirubin and HDL cholesterol levels after adjusting for age and sex. Triglyceride concentrations showed a significantly positive relationship with fetuin-A levels (*r* = 0.361, *P* < 0.001) in the nonobesity group.

### 3.3. Multiple Logistic Regression Analysis for the Risk of MA according to the Tertiles of Fetuin-A Concentrations

Multiple logistic regression analysis was performed with the risk of MA as a dependent variable; odds ratios (ORs) and 95% confidence intervals (CIs) were calculated before and after adjusting for various confounding variables (Table 3). In a model adjusted for age, sex, and BMI, individuals in the higher tertiles of fetuin-A levels exhibited an increased risk of MA compared to those in the lowest tertile in the nonobesity group (*P* for trend = 0.002), although this tendency did not reach statistically significant levels in the obesity group (*P* for trend = 0.056). However, the models achieved statistical significance in both nonobesity and obesity groups after further adjusting for smoking, alcohol consumption, and eGFR. Moreover, after multiple adjustments for diverse confounding factors, the ORs were 12.75 (95% CIs = 1.62-100.49; *P* for trend = 0.017) and 3.77 (95% CIs = 1.13-12.60; *P* for trend = 0.028) for the highest tertiles of fetuin-A compared to the lowest tertiles in groups without and with obesity, respectively. In addition, the risks for MA were stepwisely elevated along with increasing fetuin-A tertiles in each sex with no significant interactions of sex (Supplemental Table 1). There was also a significant relationship between fetuin-A level and MA in participants aged from 40 to 60 years. However, probably due to fewer participants, fetuin-A level did not show a significant relationship with MA in participants aged 60 years and above (Supplemental Table 1).

## 4. Discussion

The present study first demonstrated that hepatokines such as fetuin-A influence metabolic health in both Korean adults with and without obesity. Our results suggest that this hepatokine may underlie a novel mechanism that connects NAFLD with metabolic health phenotypes.

The prevalence of obesity has rapidly increased and has nearly doubled worldwide between 1980 and 2008 [24]. Obesity contributes to an increased risk for metabolic diseases, including type 2 diabetes, dyslipidemia, hypertension, and CVD. Although obesity is usually defined using BMI, this method has limitations, particularly in individuals with high muscle to fat ratios and in Asians [1]. A recent study identified a group of obesity, in both men and women, who have fewer cardiometabolic risk factors than expected, given their obesity status as defined by BMI. This MHO group represents between 10% and 45% of the adult population with obesity, according to the specific definition and study population [25]. MHO might be more prevalent in women than in men, and the prevalence of MHO decreases with aging in both men and women [26]. In a population-based cohort study, MHO did not increase the risk for myocardial infarction during 12 years of follow-up [27]. Moreover, Ortega et al. reported that individuals with the MHO phenotype had a lower risk of all-cause mortality as well as cardiovascular and cancer mortality compared to those with the MAO phenotype [28]. However, there have been controversies about the prognosis of MHO individuals. Kuk and Ardern showed that obesity, even in the absence of overt metabolic derangement, is related to increased all-cause mortality [29]. In the San Antonio Heart Study, the risk of developing diabetes and CVD is significantly increased in MANW and MHO individuals, respectively [30]. In a cohort of 3052 Spanish subjects, the initial prevalence of MHO was 20.8%, and 49.2% of MHO subjects transitioned to the metabolically unhealthy phenotype during a 10-year follow-up [31]. Multivariate analysis demonstrated that changes in BMI and waist circumference were positively associated with this transition [31].

Although an exact mechanism to explain metabolic health is not fully defined yet, inflammation, insulin sensitivity, physical activity, visceral fat accumulation, and hormonal changes might be important determining factors for MHO and MANW phenotypes [32]. We recently found that sleep duration is independently associated with metabolic health phenotypes in a Korean population using the data from the Korean National Health and Nutrition Examination Survey (KNHNES) [33]. Growing attention has been focused on liver fat content as an additional marker in the context of metabolic health, since the prevalence of NAFLD is significantly lower in individuals with MHO compared to those with MAO [9]. Although the MHO group had an increased risk of diabetes compared with the MHNW group, this risk was attenuated after adjustment for fatty liver [12].

Adipose tissue is a key regulator of insulin resistance and inflammation, which is involved in the development of MetS, type 2 diabetes, and CVD. Adipokines, such as leptin, adiponectin, and resistin, regulate energy homeostasis and the degree of body fatness [25]. Aguilar-Salinas et al. reported that the MHO phenotype is associated with high adiponectin levels, which are similar to those found in subjects with normal BMI [34]. In agreement with this observation, Labruna et al. demonstrated that a high serum leptin/adiponectin ratio might be a marker of MAO independent of waist circumference and BMI [35]. In the Women's Health Initiative Observational Study, MHO women had an intermediate adipokine profile, including leptin, adiponectin, and resistin levels, between MAO and MHNW women [36]. Recent studies have shown that, like adipokines, hepatokines (which are predominantly liver-derived proteins) affect systemic metabolism and energy homeostasis [37]. Fetuin-A is a 64 kDa glycoprotein that regulates metabolic balance through crosstalk between the liver and active metabolic organs [37]. Fetuin-A directly inhibits the downstream phosphorylation cascade of the insulin signaling pathway, which is closely correlated with insulin sensitivity and glucose tolerance [38]. Interestingly, fetuin-A was identified as an adaptor protein for saturated fatty acid-mediated activation of Toll-like receptor 4 (TLR4), which may integrate insulin resistance and inflammatory signaling [39]. Jialal et al. also reported that increasing secretion of fetuin-A from adipose tissue aggravate insulin resistance and the proinflammatory state of nascent MetS by TLR2 and TLR4 [40]. Circulating fetuin-A levels are increased in obesity and are associated with obesity-related disorders such as metabolic syndrome and type 2 diabetes [16, 17]. The present study showed that circulating fetuin-A levels are positively correlated with cardiometabolic risk factors, such as BMI, waist circumference, systolic and diastolic blood pressure, and FPG and negatively associated with HDL cholesterol. In accordance with previous Chinese studies [41], the risk of MA was also significantly associated with an increment of circulating fetuin-A in Koreans after adjusting for confounding factors independent of sex and BMI.

CIMT, a risk indicator for subclinical atherosclerosis, can differ between individuals with MHO and MAO phenotypes [42]. Khan et al. demonstrated that MHO women have higher CIMT and baPWV than normal weight women [42]. Zhang et al. showed that hepatic fat is a determinant of metabolic phenotypes and increased CIMT in obese adults [43]. Recently, Sung et al. demonstrated that MHO subjects had a higher risk of fatty liver but did not have an increased risk of preclinical atherosclerosis [44]. In contrast, MAO and MANW groups are at risk for fatty liver and preclinical atherosclerosis [44]. In a systemic review, Roberson et al. concluded that MHO is an important phenotype with an intermediate CVD risk between MHNW and MAO [45]. In our previous study, subjects with the MANW phenotype exhibited an increased carotid atherosclerosis and arterial stiffness compared to those with MHNW or MHO [18]. In this study, the MA group showed significantly increased baPWV values in both subjects with and without obesity; however, CIMT values did not differ significantly based on MH phenotypes. Considering that baPWV values represent arterial stiffness in the early atherosclerotic process, our results suggest that baPWV may more sensitively reflect the subtle effects of a MA phenotype.

There are several limitations to this study. First, this was a cross-sectional analysis that does not allow us to draw causal conclusions. Secondly, because the study included only Asian subjects, the results cannot be extrapolated to other ethnic populations. The results from our study need to be confirmed in other ethnicities in the future.

## 5. Conclusion

This study presents the novel finding that fetuin-A, a representative hepatokine, is correlated with metabolic health phenotypes, such as MHO and MANW, as well as arterial stiffness and various cardiometabolic parameters in Korean men and women. These results suggest that fetuin-A may have an important role in determining and predicting metabolic health and consequences independent of BMI.

## Figures and Tables

**Table 1:** Characteristics of study subjects based on obesity and metabolic health phenotype.

	Nonobese				Obese	
	MHNW (*N* = 145)	MANW (*N* = 23)	*P*	MHO (*N* = 59)	MAO (*N* = 63)	*P*
Age (years)	53 (48, 57)	55 (52, 57)	0.165	53.0 ± 5.8	52.7 ± 6.0	0.778
Sex (M : F)	91 : 54	13 : 10	0.567	41 : 18	45 : 18	0.815
Waist circumference (cm)	76.4 ± 7.0	82.0 ± 5.0	<0.001	86 (82, 91)	90 (87, 94)	<0.001
Current smoker (*n*, %)	28 (19.3)	8 (34.8)	0.105	9 (15.3)	18 (28.6)	0.077
Alcohol (*n*, %)	79 (54.5)	10 (43.5)	0.326	32 (54.2)	33 (52.4)	0.837
SBP (mmHg)	113 (103, 122)	125 (117, 141)	<0.001	118.6 ± 13.2	127.7 ± 14.0	<0.001
DBP (mmHg)	74.6 ± 9.5	82.7 ± 9.8	<0.001	78.5 ± 9.9	83.4 ± 11.1	0.012
Hemoglobin (g/dL)	14.1 ± 1.4	14.4 ± 1.3	0.353	14.3 (13.4, 15.0)	14.8 (13.7, 15.4)	0.065
WBC (10^3^/*μ*L)	5.3 (4.4, 6.3)	5.9 (5.1, 7.0)	0.034	6.0 (4.8, 6.8)	6.1 (5.4, 7.2)	0.249
Platelet count (10^3^/*μ*L)	206 (184, 229)	239 (190, 262)	0.033	211.7 ± 48.3	219.6 ± 48.0	0.367
BUN (mg/dL)	14.2 (12.4, 17.0)	13.6 (12.5, 16.5)	0.949	15.0 (12.2, 18.3)	15.5 (11.9, 16.9)	0.417
Creatinine (mg/dL)	0.8 (0.6, 0.9)	0.7 (0.6, 0.9)	0.693	0.8 (0.6, 0.9)	0.8 (0.7, 0.9)	0.307
eGFR (mL/min/1.73 m^2^)	102.7 (95.8, 107.8)	101.1 (95.4, 105.9)	0.124	100.2 (95.9, 104.9)	96.6 (89.7, 106.4)	0.252
Total bilirubin (mg/dL)	0.7 (0.6, 1.0)	0.7 (0.5, 1.1)	0.782	0.8 (0.6, 0.9)	0.7 (0.6, 0.9)	0.234
Albumin (g/dL)	4.4 (4.2, 4.5)	4.4 (4.2, 4.5)	0.247	4.3 (4.3, 4.5)	4.4 (4.3, 4.5)	0.027
AST (IU/L)	23 (20, 31)	27 (21, 36)	0.128	26 (22, 34)	29 (23, 43)	0.038
ALT (IU/L)	19 (15, 27)	27 (23, 38)	0.004	23 (17, 38)	32 (20, 50)	0.030
hsCRP (mg/L)	4 (2, 12)	11 (3, 19)	0.113	6 (3, 17)	9 (4, 23)	0.259
Glucose (mg/dL)	95 (88, 101)	105 (99, 109)	<0.001	95 (91, 99)	106 (101, 126)	<0.001
LDL cholesterol (mg/dL)	116 (100, 141)	138 (111, 158)	0.010	134 (108, 155)	109 (83, 145)	0.004
HDL cholesterol (mg/dL)	55 (46, 67)	45 (37, 50)	<0.001	51 (44, 58)	44 (37, 50)	0.001
Triglyceride (mg/dL)	87 (63, 125)	164 (95, 196)	<0.001	111 (74, 147)	187 (122, 269)	<0.001
Mean baPWV (m/sec)	13.1 (12.0, 14.4)	13.9 (13.0, 16.8)	0.005	13.1 (11.9, 14.3)	13.7 (12.8, 15.4)	0.025
Mean CIMT (mm)	0.7 (0.6, 0.7)	0.7 (0.6, 0.7)	0.392	0.7 (0.6, 0.7)	0.7 (0.6, 0.7)	0.904

MHNW: metabolically healthy normal weight; MANW: metabolically abnormal but normal weight; MHO: metabolically healthy obesity; MAO: metabolically abnormal obesity; SBP: systolic blood pressure; DBP: diastolic blood pressure; WBC: white blood cell count; BUN: blood urea nitrogen; eGFR: estimate glomerular filtration rate; AST: aspartate aminotransferase; ALT: alanine aminotransferase; hsCRP: high-sensitivity C-reactive protein; LDL: low-density lipoprotein; HDL: high-density lipoprotein; baPWV: brachial-ankle pulse wave velocity; CIMT: carotid intima-media thickness.

**Table 2:** Spearman correlation analysis between fetuin-A concentrations and various cardiometabolic risk parameters (age, sex-adjusted).

	Total		Nonobesity		Obesity	
	*r*	*P*	*r*	*P*	*r*	*P*
BMI (kg/m^2^)	0.217	<0.001	0.101	0.194	0.062	0.504
Waist circumference (cm)	0.272	<0.001	0.199	0.010	0.141	0.124
Current smoker (%)	−0.006	0.913	0.026	0.743	−0.021	0.820
Alcohol (%)	−0.093	0.116	−0.079	0.314	−0.121	0.187
SBP (mmHg)	0.132	0.025	0.110	0.157	0.026	0.780
DBP (mmHg)	0.149	0.012	0.153	0.049	0.058	0.526
Hemoglobin (g/dL)	0.002	0.967	−0.077	0.325	0.112	0.224
WBC (10^3^/*μ*L)	0.158	0.007	0.141	0.070	0.096	0.298
Platelet count (10^3^/*μ*L)	0.108	0.068	0.206	0.008	−0.043	0.64
BUN (mg/dL)	0.006	0.920	−0.019	0.812	0.005	0.958
Creatinine (mg/dL)	0.104	0.078	0.169	0.030	−0.041	0.653
eGFR (mL/min/1.73 m^2^)	−0.087	0.143	−0.152	0.051	0.061	0.510
Total bilirubin (mg/dL)	−0.153	0.009	−0.209	0.007	−0.071	0.443
Albumin (g/dL)	0.098	0.096	0.087	0.264	0.062	0.501
AST (IU/L)	0.099	0.096	−0.019	0.811	0.188	0.040
ALT (IU/L)	0.182	0.002	0.071	0.364	0.229	0.012
hsCRP (mg/L)	0.054	0.361	0.067	0.390	−0.083	0.370
Glucose (mg/dL)	0.189	0.001	0.132	0.091	0.202	0.027
LDL cholesterol (mg/dL)	−0.043	0.467	−0.015	0.848	−0.121	0.188
HDL cholesterol (mg/dL)	−0.196	0.001	−0.165	0.034	−0.141	0.124
Triglyceride (mg/dL)	0.322	<0.001	0.361	<0.001	0.156	0.089
Mean baPWV (m/sec)	0.062	0.294	0.113	0.148	−0.039	0.676
Mean CIMT (mm)	0.066	0.269	0.051	0.514	0.053	0.566

BMI: body mass index; SBP: systolic blood pressure; DBP: diastolic blood pressure; WBC: white blood cell count; BUN: blood urea nitrogen; eGFR: estimate glomerular filtration rate; AST: aspartate aminotransferase; ALT: alanine aminotransferase; hsCRP: high-sensitivity C-reactive protein; LDL: low-density lipoprotein; HDL: high-density lipoprotein; baPWV: brachial-ankle pulse wave velocity; CIMT: carotid intima-media thickness.

**(a):** 

	T1	T2	T3	*P* for trend
No of cases/references	2/54	8/48	13/43	
Cutoff of fetuin-A (*μ*g/mL)	330.7-544.2	545.0-679.3	679.4-1316.8	
Model 1	1	3.91 (0.72, 21.34)	10.81 (2.05, 56.96)	0.002
Model 2	1	4.95 (0.78, 31.40)	10.03 (1.66, 60.81)	0.009
Model 3	1	9.14 (1.21, 69.05)	12.90 (1.83, 90.80)	0.011
Model 4	1	8.30 (1.08, 63.72)	12.75 (1.62, 100.49)	0.017

**(b):** 

	T1	T2	T3	*P* for trend
No of cases/references	15/25	24/17	24/17	
Cutoff of fetuin-A (*μ*g/mL)	372.5-594.2	595.5-764.5	784.3-1572.3	
Model 1	1	2.57 (1.00, 6.59)	2.53 (0.96, 6.66)	0.056
Model 2	1	3.48 (1.26, 9.64)	2.99 (1.07, 8.37)	0.036
Model 3	1	4.48 (1.43, 14.06)	3.44 (1.06, 11.18)	0.036
Model 4	1	4.22 (1.32, 13.52)	3.77 (1.13, 12.60)	0.028

Model 1: adjusted for age, sex, and body mass index (BMI); model 2: adjusted for model 1 + smoking, alcohol, and estimate glomerular filtration rate (eGFR); model 3: adjusted for model 2 + aspartate aminotransferase (AST), alanine aminotransferase (ALT), total bilirubin, and albumin; model 4: adjusted for model 3 + white blood cell count, hemoglobin, and platelet count.

## Data Availability

The datasets analyzed during the current study are not publicly available due to the consent agreements signed by the participants but are available on reasonable request after agreement of IRB and participants.

## Supplementary Materials

Supplementary Materials Supplemental Table 1: relative risk of metabolic abnormal in subgroups according to tertiles of fetuin-A concentrations.
