# Supplementary material for: Relationship of Circulating Fetuin-A Levels with Body Size and Metabolic Phenotypes
Source: Int J Endocrinol. 2018 Dec 24;2018:7918714. doi: 10.1155/2018/7918714 (PMC6323440; doi:10.1155/2018/7918714)
Supplement: Supplementary Materials — Supplemental Table 1: relative risk of metabolic abnormal in subgroups according to tertiles of fetuin-A concentrations. [file 7918714.f1.docx]

|  | T1 | T2 | | T3 | *P* for trend | *P* for interaction |
| --- | --- | --- | --- | --- | --- | --- |
| No | 97 | 96 | | 97 |  |  |
| **Unadjusted** |  |  | |  |  |  |
| **Sex** |  |  | |  |  |  |
| Male (n=190) | 1 | 2.51 (1.13, 5.58) | | 4.04 (1.76, 9.27) | 0.001 | 0.770 |
| Female (n=100) | 1 | 2.09 (0.47, 9.41) | | 5.39 (1.41, 20.6) | 0.014 |  |
| **Age (years)** |  |  |  | |  |  |
| 40-60 (n=254) | 1 | 2.18 (1.01, 4.71) | | 5.09 (2.41, 10.726) | <0.001 | 0.036 |
| ≥60 (n=36) | 1 | 6.67 (0.96, 46.56) | | 0.50 (0.05, 5.51) | 0.571 |  |
| **Adjusted*** |  |  | |  |  |  |
| **Sex** |  |  | |  |  |  |
| Male (n=190) | 1 | 3.14 (1.11, 8.90) | | 3.92 (1.31, 11.69) | 0.015 | 0.816 |
| Female (n=100) | 1 | 4.49 (0.66, 30.50) | | 7.47 (1.30, 42.79) | 0.024 |  |
| **Age (years)** |  |  | |  |  |  |
| 40-60 (n= 254) | 1 | 3.20 (1.13, 9.10) | | 6.92 (2.42, 19.81) | <0.001 | 0.008 |
| ≥60 (n=34) | 1 | 17.13 (1.53, 192.44) | | 0.19 (0.01, 3.86) | 0.276 |  |

Supplemental Table 1. Relative risk of metabolic abnormal in subgroups according to tertiles of fetuin-A concentrations

*Adjusted for age, sex, body mass index (BMI), smoking, alcohol consumption, estimate glomerular filtration rate (eGFR), aspartate aminotransferase (AST), alanine aminotransferase (ALT), total bilirubin, albumin, white blood cell count, hemoglobin, and platelet count
